# Making sense of Wnt signaling—linking hair cell regeneration to development

**DOI:** 10.3389/fncel.2015.00066

**Published:** 2015-03-11

**Authors:** Lina Jansson, Grace S. Kim, Alan G. Cheng

**Affiliations:** Department of Otolaryngology-Head and Neck Surgery, School of Medicine, Stanford UniversityStanford, CA, USA

**Keywords:** beta-Catenin, Lgr5, Axin2, supporting cells, cochlea, planar cell polarity

## Abstract

Wnt signaling is a highly conserved pathway crucial for development and homeostasis of multicellular organisms. Secreted Wnt ligands bind Frizzled receptors to regulate diverse processes such as axis patterning, cell division, and cell fate specification. They also serve to govern self-renewal of somatic stem cells in several adult tissues. The complexity of the pathway can be attributed to the myriad of Wnt and Frizzled combinations as well as its diverse context-dependent functions. In the developing mouse inner ear, Wnt signaling plays diverse roles, including specification of the otic placode and patterning of the otic vesicle. At later stages, its activity governs sensory hair cell specification, cell cycle regulation, and hair cell orientation. In regenerating sensory organs from non-mammalian species, Wnt signaling can also regulate the extent of proliferative hair cell regeneration. This review describes the current knowledge of the roles of Wnt signaling and Wnt-responsive cells in hair cell development and regeneration. We also discuss possible future directions and the potential application and limitation of Wnt signaling in augmenting hair cell regeneration.

## The Wnt signaling pathway

Wnts (Wingless-related integration site) are a set of secreted factors that together with the Frizzled receptors make up the basis of the Wnt signaling pathway. Components of the pathway were discovered 30 years ago by different researchers working simultaneously in mouse and *Drosophila* models (Nusse et al., 1984; Cabrera et al., 1987; Rijsewijk et al., 1987), and these seminal discoveries have since sparked multiple lines of research to further elucidate the many branches and functions of this signaling pathway. Today, Wnt signaling is known to regulate stem cell pluripotency as well as many processes during development such as segmentation, polarization, cell proliferation, specification and differentiation (Logan and Nusse, 2004).

**Figure 1:**
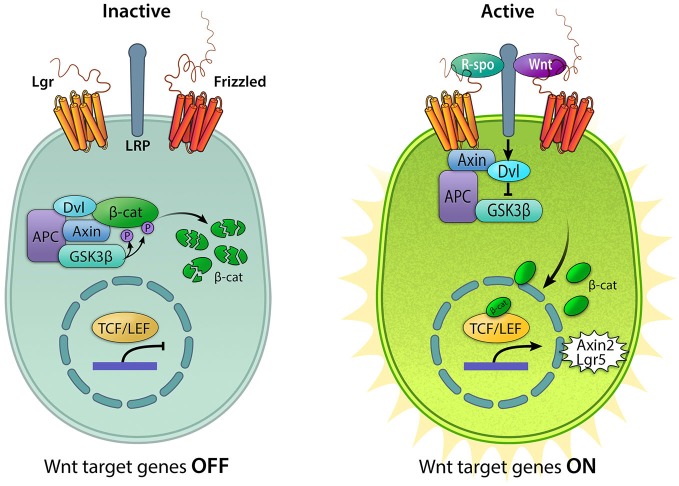
**Active and inactive Wnt/β-catenin signaling**. In the absence of Wnt ligands, the destruction complex consisting of Axin, APC, GSK3β, and Dvl (Adenomatous polyposis coli, Glycogen synthase kinase 3β, and Disheveled) resides in the cytoplasm where it binds to and phosphorylates β-catenin (β-cat), leading to its degradation. In this “off” state, T cell factor/lymphoid enhancer-binding factor (TCF/LEF) is inactive due to its interaction with the repressor Groucho. The pathway is activated upon binding of Wnt ligands to the Frizzled receptors and the co-receptor lipoprotein receptor-related protein (LRP) 5/6, resulting in the sequestration of Axin, recruitment of Disheveled, and the disintegration of the destruction complex. Binding of R-spondins (R-spo) to Lgr4/5/6 receptor stabilizes Frizzled. Accumulation of cytoplasmic β-catenin allows it to translocate into the nucleus and bind the TCF/LEF family of transcription factors to upregulate Wnt target genes, including *Axin2* and *Lgr5*.

Wnts are glycosylated proteins that usually act locally on neighboring cells or on the Wnt-secreting cells themselves. There are 19 separate *Wnt* genes in the human and murine genome, 15 in the zebrafish and 8 in *Drosophila* (Miller et al., 1999). The target cell expresses a Frizzled receptor as well as the co-receptor LRP5/6. Upon Wnt ligand binding, LRP5/6 is brought in complex with the Wnt-bound Frizzled receptor. This triggers the activation of Disheveled (Dvl) and the dismantling of a complex consisting of glycogen synthase kinase 3β (GSK3β), adenomatosis polyposis coli (APC) and Axin (Figure 1). In a model where the pathway is simplified in “on-off” states, the transcriptional co-regulator β-catenin is continually targeted for proteasomal degradation by the GSK3β/APC/Axin complex when Wnt ligands are absent and the pathway is inactive. In the presence of bound Wnt ligands, degradation is prevented and β-catenin is free to translocate to the nucleus and combine with transcription factors TCF/LEF to initiate the transcription of Wnt target genes (Logan and Nusse, 2004).

Wnt signaling is known to regulate a wide range of developmental processes such as body axis and segment polarity, mesoderm and endoderm differentiation, hair follicle and nephron development, and epithelial-mesenchymal transition (Logan and Nusse, 2004). As with many other pathways that are important for rapid cell division and cell migration during development, Wnt signaling is associated with cancer formation and several components of the pathway are known oncogenes and tumor suppressors (Ring et al., 2014). Similarly, many of the pro-proliferative genes involved in development have also been revealed to govern stem cell pluripotency (Karamboulas and Ailles, 2013; Sui et al., 2013). Thus, it is not surprising that an emerging body of work links Wnt signaling to stem cell potency and regeneration in several adult tissues.

In the inner ear, the auditory organ houses mechanosensitive hair cells required for translating sound vibration to electric impulses. The vestibular organs, comprised of the semicircular canals (SSCs), the utricle, and the saccule, also contain sensory hair cells in order to detect head position and motion. Both auditory and vestibular signals are in turn relayed centrally via the spiral and vestibular ganglion neurons, allowing for sound and balance perception. Although the mature mammalian cochlea is a terminally differentiated, non-regenerating organ, regeneration has been described in the auditory systems of lower vertebrates and additionally to a limited extent in the mammalian vestibular organs. Numerous studies have characterized the multiple roles of the Wnt signaling during cochlear development. Moreover, recent studies have highlighted potential applications of Wnt signaling in promoting hair cell regeneration. We will begin by summarizing the work done on Wnt signaling in inner ear development.

## Otocyst and vestibular development

The inner ear derives from the otic placode. Shortly after gastrulation, molecular cues including fibroblast growth factor (FGF) signaling originating from the mesendoderm and the hindbrain instruct the preplacodal field surrounding the neural plate to form a patch of *Pax2*-expressing ectoderm, which constitutes the otic-epibranchial placodes (Noramly and Grainger, 2002; Brown et al., 2003; Ladher et al., 2010; Padanad and Riley, 2011). Subsequently, the attenuation of FGF signaling along with high levels of Wnt signaling from the hindbrain coordinate the specification of otic fate in the medial *Pax2*-expressing area. Conversely, low levels of Wnt signaling, coupled with FGF signaling, promote epibranchial fate laterally (Freter et al., 2008). Ohyama et al. showed that Wnt signaling is active within the otic placode and that Wnt inhibition via β-*catenin* deletion leads to an expansion of the epibranchial domains at the expense of the otic placodal cells. When β-catenin is instead stabilized to increase canonical Wnt signaling activity, otic ectoderm expands at the expense of epibranchial cells (Ohyama et al., 2006). Therefore, Wnt/β-catenin signaling is required for the specification of the otic placode size by restricting the otic lineage to a subset of *Pax2*-positive placodal cells that will go on to form the otocyst (Jayasena et al., 2008; Freyer and Morrow, 2010; Mahoney Rogers et al., 2011).

**Figure 2:**
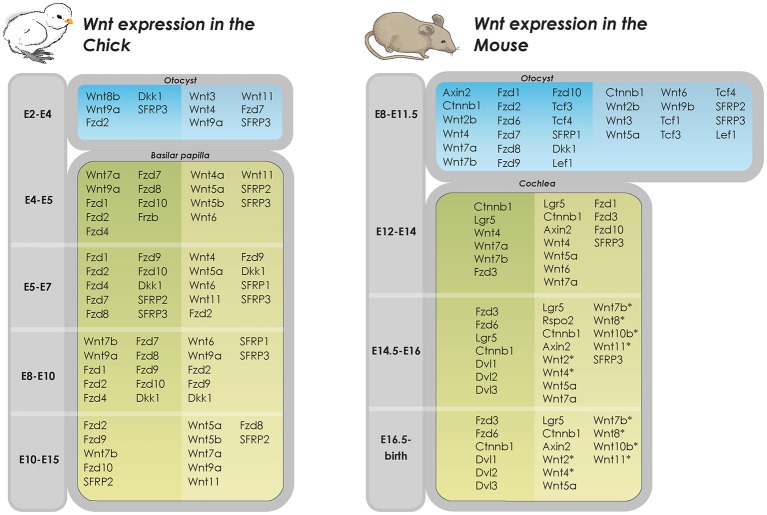
**Wnt expression in developing inner ear in chicken and mice**. Overview of Wnt gene expression in the developing inner ear (Riccomagno et al., 2005; Noda et al., 2012; Vendrell et al., 2013). Dark shaded columns on the left represent epithelium of otic cyst and lighter shaded columns on the right periotic mesenchymal tissues. In later developmental states, dark shaded columns on the left represent pro-sensory and sensory areas whereas lighter shaded columns on the right represent non-sensory areas (Dabdoub et al., 2003; Wang et al., 2005; Qian et al., 2007; Etheridge et al., 2008; Sienknecht and Fekete, 2008, 2009; Rida and Chen, 2009; Chai et al., 2011; Bohnenpoll et al., 2014). *determined by PCR, specific cell type expression unknown.

Initial work on the developing chicken otic placode implicated the involvement of *Wnt8c* (mouse *Wnt8a*) in otic ectoderm specification (Ladher et al., 2000; Urness et al., 2010). However, deletion of *Wnt8c* in the developing otic placode in zebrafish only delayed, but did not prevent, otic placode development (Phillips et al., 2004). Redundancy among Wnt ligands is well established in numerous developing systems and mapping of gene expression show that most components of the pathway, including the Wnt ligands, are expressed in a strict spatio-temporal manner during chicken inner ear development (Sienknecht and Fekete, 2009; Figure 2) suggesting that the partial overlap in expression of Wnts may account for such redundancy (Logan and Nusse, 2004; Gleason et al., 2006; Sienknecht and Fekete, 2009). For example, although individual gene deletions of *Wnt1* or *Wnt8a* result in normal inner ear development, mice deficient in both *Wnt1* and *Wnt8a* exhibit disruption of the dorsal patterning of the otocyst. This results in an underdeveloped endolymphatic sac while the formation of the otic placode and cochlear and vestibular sensory organs are unaffected (Vendrell et al., 2013). In addition, Wnt1 and Wnt3a have been shown to work redundantly in regulating the patterning of the dorsal otocyst. Riccomagno et al. determined that although the placode develops normally in *Wnt1*^−/−^; *Wnt3a*^−/−^ double knockout mice, absence of these Wnt members from the dorsal neural tube during later stages of otocyst development results in severe vestibular defects, including a complete lack of utricle and saccule. Intriguingly, in addition to the dorsal vestibular defects, the double mutant also displays a ventral phenotype with a hypoplastic cochlea (Riccomagno et al., 2005). The authors also found that Wnt signaling is both sufficient and necessary for inducing the expression of dorsal otic genes (*Dlx5, Gbx2*). Lastly, Wnt-responsive cells in the early otic epithelium was found to contribute to several different parts of the inner ear, including the majority of the vestibular system and parts of the cochlea. A similar finding on the effect of *Wnt3a* in dorsal-ventral patterning of the inner ear was recently described in the zebrafish (Forristall et al., 2014).

In subsequent stages of vestibular inner ear development, the endolymphatic sac and two additional evaginations, the canal pouches, extend from the dorsal otocyst. The two canal pouches generate the three SSCs through a coordinated yet complex process of fusion and resorption (Chang et al., 2004). Using whole embryo culture and defined modulators of the Wnt pathway, Noda et al. discovered that canonical Wnt signaling is required for the formation of the fusion plate where the two evaginated pouches join (Noda et al., 2012). They further showed the existence of a stepwise restriction of Wnt activity in the early SSCs and that the canonical Wnt pathway promotes an increase in cell number in the dorsal otocyst through both proliferation and inhibition of apoptosis.

Similarly, the mouse model where *ß-catenin* was selectively deleted in Wnt-responsive cells in the dorsal part of the otocyst shortly before canal pouch evagination displayed the failure of formation of two of the SSCs. In addition, vestibular hair cells and supporting cells were absent (Rakowiecki and Epstein, 2013). Conversely, elevating β-catenin levels resulted in an expansion of canal epithelium, a loss of the fusion plate, and block in resorption. Moreover, these investigators also found a dual role for canonical Wnt signaling that corresponded with the previously described sequential restriction of the Wnt-responsive area in the developing SSCs. Whereas Wnt signaling is required for the expansion and maintenance of the pouch epithelium during early SSC formation, cells in the epithelium are no longer responsive to Wnt signaling after fusion plate formation. Instead, cells located in the fusion plate require active Wnt signaling for the proper resorption and ultimately the formation of the fluid-filled SSCs (Rakowiecki and Epstein, 2013). While these gain- and loss-of-function experiments have helped reveal the myriad of roles of Wnt/β-catenin signaling during development of the vestibular organs, it is important to point out that the levels of Wnt signaling also critically regulate morphogenesis as both inhibiting and activating modulators lead to a malformed vestibular system (Stevens et al., 2003).

## Cochlear development

Active canonical Wnt signaling is mediated by the nuclear translocation of the key player, β-catenin, which complexes with transcription factors of the TCF/LEF family to initiate transcription (Figure 1). Several reporter mouse strains have been created to report on active Wnt/β-catenin signaling. These employ one or several TCF binding sites followed by either a fluorescent reporter or the enzyme beta-galactosidase to detect Wnt-responsive cells (DasGupta and Fuchs, 1999; Maretto et al., 2003; Ferrer-Vaquer et al., 2010; also reviewed in Barolo, 2006). Using a fluorescent Wnt reporter mouse, Jacques et al. showed that Wnt/β-catenin signaling is active in cochlear prosensory cells during early stages of cochlear development (Jacques et al., 2012). Reporter activity gradually diminishes until late embryonic ages when expression becomes restricted to a subset of supporting cells in the cochlear duct (Hensen’s cells, pillar cells, inner phalangeal cells and inner border cells).

To determine the functional significance of canonical Wnt signaling in prosensory cells, these authors first used small molecule inhibitors of Wnt signaling in organotypic cultures of the embryonic (E12.5) cochlea and found that inhibition of Wnt signaling results in decreased proliferation of prosensory cells (Jacques et al., 2012). Conversely, application of LiCl to activate Wnt signaling causes an expansion of the *Sox2*-expressing prosensory domain and an increase in hair cell number. Recently, Shi et al. used a transgenic approach to carry out similar gain- and loss-of-function experiments in the developing mouse cochlea *in vivo* (Shi et al., 2014). Employing an inducible mouse model to manipulate the *Sox2*-expressing prosensory domain, they found that *β-catenin* deletion prevented differentiation of both hair cells and pillar cells. When *β-catenin* deletion instead occurred after the initiation of hair cell differentiation (after the onset of early hair cell markers *Atoh1* and *Gfi1*), using a hair cell specific Cre driver, hair cell maturation appeared unaffected, suggesting that Wnt/β-catenin signaling is required for proliferation of prosensory cells and for differentiation of hair cells but not for their subsequent maintenance. In support of the previous *in vitro* results, stabilization of β-catenin before the onset of hair cell differentiation increased cell divisions in the prosensory domain and induced the formation of supernumerary hair cells in a disorganized cochlea (Shi et al., 2014). Similarly, prior work using a retroviral approach to deliver β-catenin to the developing chicken otocyst found ectopic hair cell formation (Stevens et al., 2003). On the other hand, Wnt/β-catenin activation in neonatal cochlear supporting cells led to a primarily mitogenic response and did not perturb hair cell maturation (Chai et al., 2012; Shi et al., 2013).

Another reporter of active Wnt/β-catenin signaling is *Axin2* expression, which acts as a negative feedback inhibitor of the pathway (Lustig et al., 2002). In the developing cochlea, *Axin2* is highly expressed in the periotic mesenchymal cells that surround the cochlear duct in a spatiotemporal pattern remarkably different from that of *Lgr5* and TCF/LEF reporters (Chai et al., 2011; Jacques et al., 2012). Using conditional knockout mice, Bohnenpoll et al. found that deletion of *β*-catenin in *Tbx1*-expressing periotic mesenchymal cells led to loss of *Axin2* expression, reduction in the overall cell number and subsequent differentiation into fibrocytes (Bohnenpoll et al., 2014). Similar to cells inside the cochlear duct, stabilized β-catenin in *Tbx1*-expressing cells induced a mitogenic response. This study suggests that Wnt signaling is required for correct differentiation of periotic mesenchymal cells. Our group has found *Axin2* expression in tympanic border cells, which are derivatives of periotic mesenchymal cells, in the neonatal cochlea and have shown that they can behave as hair cell progenitors (Jan et al., 2013). The exact roles of *Axin2*-expressing periotic mesenchymal cells and tympanic border cells during cochlear development are unclear.

## Non-canonical Wnt signaling: focus on PCP

**Figure 3:**
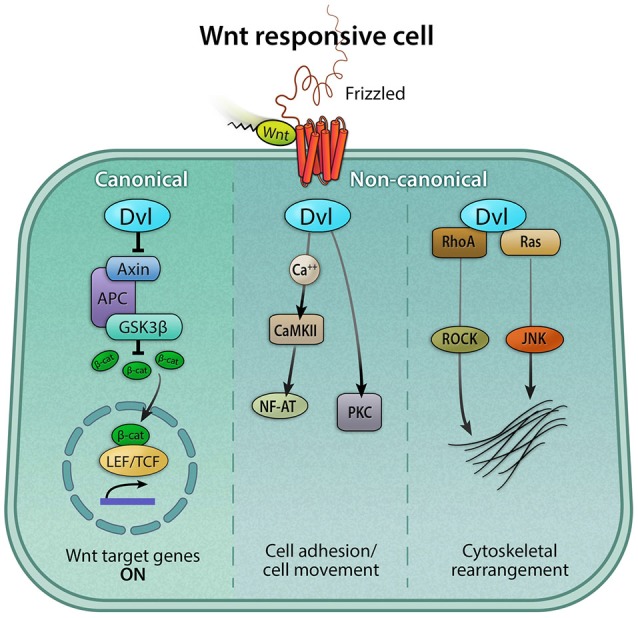
**Canonical and non-canonical Wnt signaling pathways**. The Wnt pathway can be classified broadly as canonical and non-canonical. Both pathways are activated by a Wnt ligand to the Frizzled receptor. The active canonical pathway is mediated by β-catenin, which translocates into the nucleus and it acts as a co-activator of the TCF/LEF transcription factor, leading to the upregulation of Wnt target genes. The two major non-canonical pathways are Wnt/calcium and Planar Cell Polarity (PCP) pathways. In the Wnt/calcium pathway, Wnt binding to Frizzled activates Dvl, which stimulates calcium release from the endoplasmic reticulum, activating calcium-binding proteins including protein kinase C (PKC) and calmodulin-dependent kinase II (CamKII), and in turn, the transcription factor NFAT. The Wnt/calcium pathway has been shown to regulate cell movement and axis formation during embryogenesis. The Wnt/PCP pathway is mediated by the GTPases RhoA and Ras, which, via the activation of the RhoA-Rho-associated kinase (ROCK) axis or JNK, can exert effects on the cytoskeleton.

In addition to canonical Wnt signaling, Wnt proteins can exert cellular responses in a β-catenin-independent manner via two separate non-canonical pathways (Figure 3). First, the planar-cell-polarity (PCP) pathway signals through Frizzled receptors and Disheveled to primarily rearrange the cytoskeleton, change cell morphology, and affect gene expression. Secondly, the Wnt/calcium pathway signals through phospholipase C to release intracellular calcium stores to ultimately affect genes involved in migration and cell fate (Gómez-Orte et al., 2013).

The mammalian organ of Corti represents one of the most organized epithelial structures. Nonsensory supporting cells and sensory hair cells are interlaced in a precise pattern to form the organ of Corti (OC), which in turn is strictly organized along a basal-apical axis (Wu and Kelley, 2012). Additionally, the stereocilia on the apical surface of hair cells consists of orderly rows of actin-based projections arranged in a linear and V-shaped pattern on inner and outer hair cells (Peng et al., 2011). On the outer hair cells, each row of stereocilia increases in height, forming a distinct staircase pattern, ending with the lone kinocilium at the tip of the V-shape that is responsible for directing the PCP of hair cells during development. This precise patterning of hair bundles is repeated along the cochlea and disorganization results in perturbation of sound perception (Yoshida and Liberman, 1999; Ezan and Montcouquiol, 2013).

Dabdoub et al. demonstrated that disruption of overall Wnt activity through either directly inhibiting ligand binding or halting Wnt ligand diffusion results in a disorientation of hair cell bundles, indicating that secreted Wnt proteins are required for correct PCP in the cochlea (Dabdoub et al., 2003). The authors also found that *Wnt7a* is expressed in the developing cochlear duct and cochlear explants cultured in Wnt7a conditioned medium resulted in misalignment of hair cell bundles. However, *Wnt7a* deficient mice revealed no anomaly in bundle orientation. This indicates that Wnt7a is not required for correct bundle orientation and suggests that other Wnt members may compensate for Wnt7a or independently mediate the Wnt/PCP pathway in the developing cochlea (Dabdoub et al., 2003). Another candidate mediator of the Wnt/PCP protein is *Wnt5a*, which is expressed in the Kölliker’s organ medial to the organ of Corti (Qian et al., 2007). A Wnt antagonist, *Frzb* (also known as *SFRP3*), is expressed on the opposite side of the developing organ of Corti, possibly creating a gradient of active Wnt5a signaling across the sensory domain. In contrast to *Wnt7a* deficient mice, *Wnt5a* null mice display clear PCP defects with a slightly shortened and broader cochlea consisting of additional rows of hair cells along its entire length, especially towards the apical end. However, this phenotype is only observed in about one third of the mutant animals (Lewis and Davies, 2002; Qian et al., 2007). Subsequent studies on Frizzled receptors have complemented the findings in *Wnt5a* null mice. Both the Wnt receptor Frizzled3 and Frizzled6 are expressed in cochlear and vestibular hair cells in the inner ear, and double knockout mice for both receptors display PCP defects more penetrant in the cochlear than vestibular organs (Wang et al., 2006). Specifically, hair bundles on cochlear hair cells are overtly disoriented while bundles on hair cells in the cristae are unevenly disoriented and those in the utricle and saccule are unaffected (Wang et al., 2006). This may be attributed to different sets of Frizzled receptors mediating the Wnt/PCP pathway in the cochlear vs. vestibular organs. In the cochlea, all three members of Dvls have been implicated to mediate the Wnt/PCP pathway (Figure 3). While mutant mice deficient in *Dvl1* or *Dvl2* alone were normal, double knockouts of both *Dvl1* and *Dvl2* exhibit PCP defects (Wang et al., 2005), suggesting a functional redundancy between these 2 members. These findings contrast those of *Dvl3* null mice, which displayed PCP defects in addition to loss of outer hair cells (Etheridge et al., 2008). *Dvl3* null mice with one functional allele of *Dvl2* exhibits more severe PCP defects than *Dvl3* null animals alone, again suggesting Dvl members may complement each others. A comprehensive review of Wnt/PCP can be found in a recent review by Ezan and Montcouquiol (Ezan and Montcouquiol, 2013).

## R-spondins and Lgr receptors

An important group of ligands and receptors in stem cell biology and regeneration, the R-spondin family of ligands and leucine rich repeat (Lgr) G-coupled family of receptors, was recently linked to Wnt signaling (Jin and Yoon, 2012). R-spondins can bind to three members of Lgr receptors to regulate the strength of Wnt signaling (de Lau et al., 2011). Specifically, R-spondins are ligands for Lgr4, 5 and 6, which represent a phylogenetic subgroup of Lgr receptors (de Lau et al., 2012). In most tissues where R-spondin and Lgr’s have been studied with regards to Wnt signaling, it has been found that they act to potentiate downstream Wnt signaling (Schuijers and Clevers, 2012). It is therefore quite intriguing that the only study on R-spondins in the developing inner ear postulates a negative regulatory function on Wnt signaling. Mulvaney et al. found that *R-spondin 2*, as the only member of the family (R-spondin 1–4) present during cochlear development, is expressed in the greater epithelial ridge cells (Mulvaney et al., 2013). Studying mice with targeted mutations in *R-spondin 2*, they discovered that loss of *R-spondin 2* resulted in a continuous extra row of outer hair cells in the cochlea without causing PCP defects (Mulvaney et al., 2013). When R-spondin 2 was added to explant cultures from wildtype mice, the number of outer hair cells decreased modestly, further indicating that R-spondin 2 has the opposite effect of β-catenin activation in the developing cochlea and do not appear to potentiate canonical Wnt signaling to increase the number of hair cells (Jacques et al., 2012; Shi et al., 2014). One may speculate that either there is crosstalk between R-spondin 2 and other pathways or that Wnt signaling has a putative negative effect on outer hair cell formation in the developing cochlea, possibly through a non-canonical Wnt signaling pathway.

Out of the possible Wnt-associated R-spondin receptors, *Lgr5* is the best studied in inner ear development. Chai et al. found that *Lgr5* is expressed in the cochlear duct epithelium and lateral wall during development and that the expression becomes progressively more restricted to a subset of supporting cells (Chai et al., 2011). Mice deficient in *Lgr5* die perinatally. However, morphological studies have demonstrated that cochlear development proceeds normally, and the gross morphology of the organ of Corti is normal, indicating that *Lgr5* is dispensable for hair cell formation (Chai et al., 2011). Instead, it is *Lgr5*-positive cells in the postmitotic neonatal cochlea that have garnered attention recently as a possible source of hair cell progenitors.

## Wnt signaling in stem cell regulation and tissue regeneration

In contrast to the Wnt/Frizzled/β-catenin axis, which is evolutionarily conserved from early multicellular animals and onward, the *R-spondin/Lgr* gene families are primarily a vertebrate specific evolutionary addition. One theory put forward by Clevers et al. is that they have evolved as a result of a need for a higher regulatory input in Wnt signaling in the adult tissue stem cells frequently seen in long-lived vertebrates (Clevers et al., 2014). Using R-spondin/Lgr signaling to fine-tune the Wnt pathway output would potentially strike the fine balance between stem cell proliferation and malignant cell transformation required to sustain tissue regeneration. Whether true or not, there is a striking association between the R-spondin/Lgr axis and several types of somatic stem cells. *Lgr5* especially, has garnered much attention the last few years as a high fidelity marker of stem cells in several regenerating tissues (Schuijers and Clevers, 2012). For instance, *Lgr5* marks a subset of cells in the highly proliferative intestinal epithelium (Barker et al., 2007). As the fastest proliferating organ in adult mammals, intestinal cells are completely replaced about every 4 to 5 days (Leblond and Stevens, 1948). Using genetic lineage tracing, researchers found that *Lgr5*-positive cells could give rise to all differentiated cell types in the intestine and identified them as a type of intestinal stem cell (Barker et al., 2007). This supports a model where abrogated Wnt signaling, including depletion of co-regulator β-catenin and transcription factor TCF, results in a loss of stem cells and self-renewal in the intestine (Korinek et al., 1998; Fevr et al., 2007).

*Lgr5*-positive cells exhibiting varying degree of stem cell properties have also been found in the stomach (Barker et al., 2010), hair follicle (Jaks et al., 2008) and mammary gland (de Visser et al., 2012). In the skin, where *Lgr5*-positive cells continuously give rise to new hair follicle cells, another Lgr family receptor *Lgr6*, marks a different, Wnt-independent, stem cell population that replenishes the epidermis and sebaceous glands (Barker et al., 2010). Both *Lgr5*- and *Lgr6*-expressing cells also contribute to wound healing in the skin, while *Lgr6*-positive progeny alone are responsible for establishing long-term repair of all components in the skin. In the liver, *Lgr5*-positive cells only appear after damage when they regenerate hepatocytes and bile ducts (Huch et al., 2013b). Similarly in the pancreas, *Lgr5-positive* progenitors cells also appear after damage (Huch et al., 2013a). A common trait of these cells is the ability to generate ever-expanding cultures of Lgr5 progenitor cells responsive to Wnt proteins and R-spondins *in vitro*. The manifestation of *Lgr5-positive* cells with more limited proliferative performance has also been seen in isolated cultures of cochlear cells (Chai et al., 2012; Shi et al., 2012). These cells respond to Wnt stimulation and can act as *in vitro* hair cell progenitors.

Another member of the Wnt pathway and direct Wnt target gene frequently associated with stem cells is the negative regulator Axin2 (Figure 1; Jho et al., 2002; Lustig et al., 2002; Zeng and Nusse, 2010). Bowman et al. showed that *Axin2*-positive cells could give rise to multiple restricted neural stem cell populations during development and that these Wnt/β-catenin-responsive stem cells persisted in the adult mouse (Bowman et al., 2013). Axin2 tracing likewise labels several distinct stem cell populations in the developing and adult mammary gland (van Amerongen et al., 2012). Similar to *Lgr5* and *Lgr6*, *Axin2* also marks a population of stem cells in the skin. Axin2-positive interfollicular epidermal stem cells contribute to epidermal regeneration in a Wnt/β-catenin dependent manner (Lim et al., 2013). *Axin2-marked* tympanic border cells in the neonatal cochlea have been shown to exhibit Wnt-responsive progenitor cell characteristics (Jan et al., 2013), yet their roles during the homeostasis and regeneration of the neonatal and mature cochlea are currently unknown.

## Regeneration in fish and birds

A rich body of literature describes the temporal events during the regeneration of hair cells in non-mammalian species including birds and zebrafish (Corwin and Cotanche, 1988; Ryals and Rubel, 1988; Warchol and Corwin, 1996; Brignull et al., 2009). More recently the molecular switches during these events have begun to be unveiled. In the avian utricle and the zebrafish lateral line, hair cells are turned over, and new hair cells are produced on a continuous basis (Williams and Holder, 2000). After hair cell damage in these organs, the rate of hair cell production increases (Matsui et al., 2000; Harris et al., 2003; Ma et al., 2008). Although no steady-state hair cell turnover has been observed in the chicken auditory organ, the basilar papilla, noise or aminoglycoside damage results in robust supporting cell proliferation and hair cell replacement (Corwin and Cotanche, 1988; Ryals and Rubel, 1988). In the avian inner ear and zebrafish lateral line, the predominant mode of regeneration involves supporting cell division prior to conversion of one daughter cell to a sensory hair cell. When hair cell regeneration occurs without an antecedent mitotic event, a phenomenon termed direct transdifferentiation can be observed in the regenerating basilar papilla (Roberson et al., 2004; Duncan et al., 2006; Shang et al., 2010). Based on these studies, the process of hair cell regeneration can be categorized into two modes in the basilar papilla: mitotic regeneration and direct transdifferentiation. As direct transdifferentiation is also the primary mode of regeneration in the mammalian vestibular system, its mechanism will be discussed in a later section.

Because canonical Wnt signaling has been shown to be critical for the development of posterior lateral line neuromasts (Gamba et al., 2010; Aman et al., 2011; McGraw et al., 2011; Valdivia et al., 2011), its role during the regeneration of hair cells in the neuromasts was recently examined (Head et al., 2013; Jacques et al., 2014). Using a TOP;GFP reporter transgene (Dorsky et al., 2002), Head et al. reported a low level of active Wnt/β-catenin signaling in the homeostatic neuromasts, where proliferation and hair cell renewal are present at low levels. This is in contrast with results obtained using a different reporter of active Wnt signaling, where no Wnt activity was observed during homeostasis (Jiang et al., 2014). Overexpression of *dickkopf1b (dkk1b)*, a secreted Wnt inhibitor, further reduced this baseline level of proliferation. Conversely, pharmacologic inhibition of GSK3β (using 1-azakenpaullone) increased supporting cell proliferation in a β-catenin dependent manner, indicating that the effects are mediated by canonical Wnt signaling. After neomycin-induced hair cell loss, Wnt activity as indicated by the TOP;GFP reporter transgene increases, and this rise coincides with the increase in proliferation observed in supporting cells in the neuromasts (Head et al., 2013). As in the homeostatic neuromasts, overexpression of *dkk1b* dampened the proliferative response to hair cell damage, suggesting that Wnt signaling was required at least for the mitotic phase of regeneration. Likewise, the Wnt activators 1-azakenpaullone and LiCl, which are both GSK3β inhibitors, promoted supporting cell proliferation and increased the number of hair cells formed and the overall size of the neuromasts exceeding the level of natural regeneration (Head et al., 2013; Jacques et al., 2014). In regenerating neuromasts, both supporting cells and mantle cells proliferate in response to hair cell damage with only the former contributing to hair cell regeneration (Jones and Corwin, 1993; Harris et al., 2003; Hernandez et al., 2007; Ma et al., 2008). After drug-induced Wnt activation in both damaged and undamaged tissues, cells located in the center and the periphery of the neuromasts (where supporting cells and mantle cells reside) are competent to respond by increasing mitotic events, ultimately resulting in enhanced hair cell regeneration (Head et al., 2013; Jacques et al., 2014). Yet, the functional significance of this augmented regeneration is unclear. While these studies suggest that canonical Wnt signaling is required for the proliferative response to hair cell loss and that activating Wnt/β-catenin signaling can enhance mitotic hair cell regeneration, recent insights into putative hair cell progenitors in the zebrafish posterior lateral line and chicken utricle draw a more complex picture.

Two recent studies (Jiang et al., 2014; Steiner et al., 2014) examined the temporal expression of Wnt target genes and other components of the Wnt pathway in supporting cells and mantle cells in response to hair cell damage. Jiang et al. found that changes in expression of cell cycle genes in supporting cells and mantle cells precede those of canonical Wnt signaling. Specifically, they found that both the components of the Wnt pathway (*tcf4*, *fzd7b*, *fzd8a*, *Wnt10a*) and its overall activity as measured by a TCF/LEF-GFP reporter were downregulated during the early post-damage period. Subsequently, an increase in these components precedes active Wnt signaling (also by TCF/LEF-GFP reporter) in the late phase of regeneration. In parallel, microarray data generated by Steiner et al., who isolated mantle cells after having used copper to lesion hair cells, showed a complicated damage response consisting of upregulation of a subset of the Wnt pathway components and target genes (*Wnt3*, *Wnt7a*, *Wnt9a*) but downregulation of others (*Wnt9a*, *Axin2*, *Tcf4*, *Sfrp2*) immediately after damage (Steiner et al., 2014). Interestingly, cell cycle gene expression changes immediately following hair cell degeneration coincided with a decrease in Notch and FGF signaling (Jiang et al., 2014). These results suggest that pathways other than Wnt signaling may be responsible for initiating the regenerative response of supporting cells after hair cell loss but that active Wnt signaling is both necessary and sufficient for proliferation in later stages of hair cell regeneration.

The molecular mechanisms of supporting cell proliferation in the chick utricle have received much attention. In the regenerating chick cochlea and utricle, proliferation peaks at 48 h after ototoxic injury (Alvarado et al., 2011). When the sensory epithelium from either organ was profiled, *ß-catenin* was down-regulated at both the 0 and 24 h time points after damage before being significantly upregulated at 48 h when the peak of proliferation occurred. Furthermore, RNAi knockdown of *ß*-catenin and *Wnt4* prevented supporting cell proliferation, suggesting that Wnt/β-catenin signaling is required for at least the mitotic phase of regeneration (Alvarado et al., 2011) similar to what has been described in the zebrafish neuromasts. Addition of exogenous Wnt proteins (Wnt4 and 5a) further enhanced proliferation of supporting cells, providing additional support that Wnt/β-catenin signaling also modulates the proliferative response after hair cell injury. Using the peaks of proliferation and hair cell formation (at 48 h and 168 h post hair cell injury) as references, Ku et al. examined the dynamics of gene expression, including Wnt target genes, during hair cell regeneration in the chicken utricle *in vitro* (Ku et al., 2014). Several of the examined Wnt target genes (*Lgr5, Axin2, Klf5, Lef1*) did not exhibit significant changes prior to proliferation. As was performed in the zebrafish lateral line, an examination of the timing of mRNA expression reveals a much more complex picture of Wnt signaling during regeneration. This complexity is likely attributed to the dynamic interplay of several major pathways with canonical Wnt signaling to stepwise regulate the initiation of regeneration, proliferation, and conversion of progenitor cells towards a hair cell fate. In particular, Notch signaling has been demonstrated to limit both proliferation and hair cell differentiation via the process of lateral inhibition in zebrafish lateral line (Ma et al., 2008). Direct crosstalk between Wnt and Notch signaling is also known to occur in numerous systems during development (Hayward et al., 2008). Understanding the complex weave of pathway interactions as well as the level of activity of individual pathway will likely be necessary to make additional progress towards elucidating the complete picture, depicting the mechanisms of hair cell regeneration. While more challenging, a combinatorial approach will also likely yield a more robust degree of regeneration in the mammalian inner ear.

## Wnt signaling and mammalian hair cell regeneration

In stark contrast to the avian inner ear and the zebrafish lateral line, the mature mammalian auditory and vestibular organs do not spontaneously mount a proliferative response after hair cell degeneration. While no regeneration of cochlear hair cells have been observed, the vestibular organs non-mitotically regenerate lost hair cells to a limited extent (Forge et al., 1993; Oesterle and Campbell, 2009; Lin et al., 2011). Although the roles of canonical Wnt signaling during mammalian inner ear development have been more thoroughly examined, only recently have studies begun to shed light on its possible involvement during regeneration.

As described previously, during embryonic mouse cochlear development, active Wnt/β-catenin signaling is required for initial hair cell differentiation but not subsequent maturation and maintenance (Shi et al., 2014). Overactive Wnt signaling promotes both proliferation and ectopic hair cell formation during early embryonic development as opposed to a primarily proliferative response during late embryonic development (Jacques et al., 2012). This suggests that the effects of Wnt/β-catenin signaling shift as the cochlea matures, emphasizing its context-dependent role. In the neonatal, immature cochlea, *Lgr5* is expressed in a subset of supporting cells. When isolated and cultured *in vitro*, *Lgr5*-positive supporting cells can behave as hair cell progenitors, as defined by their limited self-renewal and ability to generate new hair cells (Chai et al., 2012; Shi et al., 2012). Indeed, the acquired proliferative behavior of isolated Lgr5-positive supporting cells require secreted Wnts and become more robust in the presence of Wnt agonists, similarly to what has been described in other organs. In a transgenic model of hair cell ablation in neonatal mice, *Lgr5*-positive cells can, to a limited degree, proliferate and regenerate hair cells after damage (Cox et al., 2014), although regenerated hair cells later degenerated, likely due to a lack of survival factors.

In addition to the mitogenic effect exerted by Wnt/β-catenin signaling, studies on cochlear development in both mice and chicken suggest that overexpression of β-catenin can induce ectopic hair cell formation (Stevens et al., 2003; Jacques et al., 2012; Shi et al., 2014). When β-catenin was stabilized in supporting cells in the neonatal cochlea, a subset of supporting cells proliferated. In addition to the proliferation, a subset of supporting cells was also found to acquire a hair cell fate as demonstrated by *Atoh1* expression (Chai et al., 2012; Shi et al., 2012, 2013). Unfortunately, in the undamaged adult mouse cochlea, β-catenin stabilization fails to induce either supporting cell proliferation or hair cell formation (Shi et al., 2013). Hence, both the ability to mount a proliferative response and the capacity to acquire a hair cell fate in response to Wnt overactivation decreases and becomes spatially more restricted in the neonatal cochlea compared to its embryonic counterpart.

In contrast to the mature mammalian cochlea, the adult vestibular utricle retains a limited capacity for hair cell regeneration (Forge et al., 1993; Warchol et al., 1993). Using a transgenic hair cell ablation model in adult mice, Golub et al. estimated the extent of hair cell regeneration to be 17% over a 6 month period (Golub et al., 2012). The mode of regeneration is assumed to be direct transdifferentiation as almost no mitotic events were detected. While the function of canonical Wnt signaling has not been thoroughly examined in the mature utricle, select studies have instead examined the neonatal utricle, where supporting cells can proliferate and regenerate lost hair cells after damage (Burns et al., 2012). Without damage, pharmacologic inhibition of GSK3β enhanced supporting cell proliferation *in vitro* (Lu and Corwin, 2008).

Unlike in the cochlea, recent work from our laboratory show that *Lgr5* expression is not detected in the neonatal utricle. However, hair cell damage at that age results in an up-regulation of *Lgr5* expression in supporting cells prior to hair cell regeneration. Lineage tracing experiments further demonstrated that *Lgr5*-positive supporting cells can mitotically regenerate hair cells and that β-catenin stabilization augmented both the mitotic response and the extent of hair cell regeneration (Wang et al., in press). Taken together, the neonatal mouse cochlea and utricle both contain supporting cells that are competent to proliferate and form hair cells in response to Wnt overactivation. It is important to point out that the supporting cells in the neonatal utricle, unlike those in the adult organ, are capable of re-entering the cell cycle before hair cell regeneration. One may conceive these neonatal supporting cells as less mature, but the factors differentiating them from those residing in the adult utricle are yet to be revealed.

**Figure 4:**
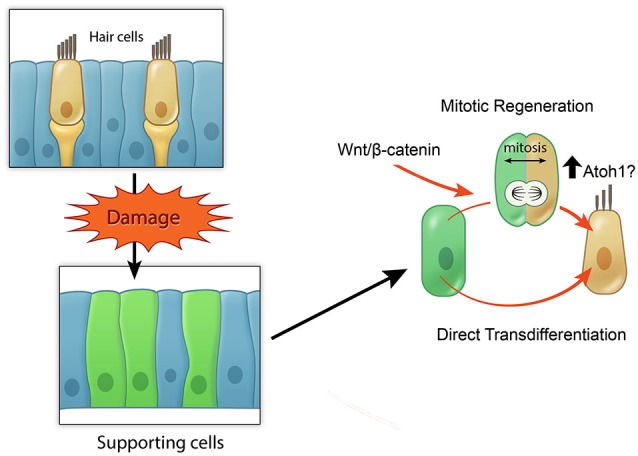
**Model of potential roles of active Wnt signaling in hair cell regeneration**. Schematic broadly depicting hair cell damage can activate supporting cells to proliferate and regenerate lost hair cells (mitotic regeneration), or to directly acquire a hair cell fate. Wnt/β-catenin signaling can increase mitotic regeneration by promoting cell cycle re-entry and also possibly by increasing Atoh1 expression.

In summary, these studies in regenerating sensory organs suggest that active Wnt/β-catenin signaling can increase the extent of mitotic hair cell regeneration (Figure 4), yet the ability of activating Wnt/β-catenin signaling alone to renew proliferation and hair cell regeneration observed in the neonatal, immature mammalian cochlea appears limited in the mature, mammalian cochlea. Considering the dynamic changes of other signaling pathways during regeneration of the zebrafish lateral line system and the chick utricle, it will be critical to understand how other signals may act in concert with Wnt signaling to regulate the process of regeneration.

## Future directions

A major hurdle in dissecting the functions of individual Wnt ligands and Frizzled receptors is their redundancy. This was illustrated previously by the lack of inner ear phenotypes in mice deficient in either *Wnt1* or *Wnt3*, yet double knockouts exhibit overt abnormal development of the otocyst. Insights into the mechanisms of packaging and secretion of Wnt proteins have introduced new approaches to overcome this hurdle. In Wnt secreting cells, Wnt proteins first undergo lipid modification, which is necessary for their activity (Willert et al., 2003), before being secreted. Studies in *Drosophila* have characterized Porcupine and Wntless to be essential for these steps, and their deficiency leads to phenotypes similar to those of Wingless (homolog of Wnt) mutants (van den Heuvel et al., 1993; Kadowaki et al., 1996; Banziger et al., 2006). Thus future studies manipulating Wnt secretion may help delineate the complex functions of secreted Wnts in the context of canonical and non-canonical Wnt signaling in the inner ear.

The identity of the cells targeted by Wnt secretions present additional obstacles. The majority of work on downstream Wnt signaling has so far focused on manipulating β-catenin through genetic approaches or by means of small molecules targeting its destruction complex. While this has garnered substantial information on where active canonical Wnt signaling is required during development and regeneration, much of the identity of Wnt secreting cells and what combinations of Wnt and Frizzled members are responsible for activating Wnt/β-catenin signaling in specific developmental and regenerative processes still remain unknown (Figure 2). How can prosensory cells, which are regulated by canonical Wnt/β-catenin signaling differentiate into hair cells whose orientation is tightly regulated by the non-canonical Wnt/PCP pathway just a few days later? Do different axes of Wnt-Frizzled operate the two pathways? Due to its striking phenotype in the ear, and especially the mammalian cochlea, the PCP pathway has been the most studied of the non-canonical Wnt signaling branches. However, the molecular response in Wnt/PCP-responsive cells upon Wnt stimulation is still poorly understood. Additionally, the role of Wnt/calcium pathway and whether it serves different or overlapping functions are unknown and actively being investigated. Furthermore, while the focus on the function of β-catenin has been in the context of canonical Wnt signaling, it is important to also consider its role in cell adhesion as it has been shown to interact closely with cadherin family members (Nelson and Nusse, 2004). Thus understanding the identity of Wnt-secreting and Wnt-responsive cells as well as the downstream mechanisms regulating their functions during hair cell development is critical in guiding our approach to incorporating Wnt manipulation during hair cell regeneration.

As previously mentioned, much work is still needed to improve our understanding of the relationships among multiple signaling pathways. The studies undertaken in species where hair cells naturally regenerate as part of homeostatic turn-over or in response to damage have begun to paint a picture of the spatio-temporal activity of pathways during supporting cell proliferation and hair cell differentiation. Some parts of the interaction of Wnt signaling with other pathways have already begun to unravel. For example, Wnt activation has been discovered to result in the upregulation of Jagged1, a member of the Notch pathway with a prosensory function (Morrison et al., 1999). Inhibition of either the Notch or Wnt pathway at early cochlear development results in diminished prosensory regions and prevented hair cell differentiation, suggesting that the pathways may intersect at least at this developmental stage (Brooker et al., 2006; Kiernan et al., 2006). Moreover, Bramhall et al. found that Notch inhibition increased hair cell regeneration by *Lgr5*-positive cells in the neonatal cochlea *in vitro* (Bramhall et al., 2014), implying that Wnt-responsive cells are prevented by Notch signaling to differentiate into hair cells. Important next steps should include mapping of the activity of such pathways in the quiescent, non-regenerating auditory epithelium and the understanding of the native competence of individual supporting cells, such as the *Lgr5*-positive cells, as the auditory and vestibular organs age. Such an understanding and the development of additional genetic and molecular tools to facilitate a combinatorial approach are critical next steps towards further facilitating mammalian hair cell regeneration.

## Conflict of interest statement

The authors declare that the research was conducted in the absence of any commercial or financial relationships that could be construed as a potential conflict of interest.

## References

[B1] Alvarado D. M. Hawkins R. D. Bashiardes S. Veile R. A. Ku Y. C. Powder K. E.. (2011). An RNA interference-based screen of transcription factor genes identifies pathways necessary for sensory regeneration in the avian inner ear. J. Neurosci. 31, 4535–4543. 10.1523/JNEUROSCI.5456-10.2011 21430154 PMC3086586

[B2] Aman A. Nguyen M. Piotrowski T. (2011). Wnt/β-catenin dependent cell proliferation underlies segmented lateral line morphogenesis. Dev. Biol. 349, 470–482. 10.1016/j.ydbio.2010.10.022 20974120

[B3] Banziger C. Soldini D. Schutt C. Zipperlen P. Hausmann G. Basler K. (2006). Wntless, a conserved membrane protein dedicated to the secretion of Wnt proteins from signaling cells. Cell 125, 509–522. 10.1016/j.cell.2006.02.049 16678095

[B4] Barker N. Huch M. Kujala P. van de Wetering M. Snippert H. J. van Es J. H.. (2010). Lgr5(+ve) stem cells drive self-renewal in the stomach and build long-lived gastric units in vitro. Cell Stem Cell 6, 25–36. 10.1016/j.stem.2009.11.013 20085740

[B5] Barker N. van Es J. H. Kuipers J. Kujala P. van den Born M. Cozijnsen M.. (2007). Identification of stem cells in small intestine and colon by marker gene Lgr5. Nature 449, 1003–1007. 10.1038/nature06196 17934449

[B6] Barolo S. (2006). Transgenic Wnt/TCF pathway reporters: all you need is Lef? Oncogene 25, 7505–7511. 10.1038/sj.onc.1210057 17143294

[B7] Bohnenpoll T. Trowe M. O. Wojahn I. Taketo M. M. Petry M. Kispert A. (2014). Canonical Wnt signaling regulates the proliferative expansion and differentiation of fibrocytes in the murine inner ear. Dev. Biol. 391, 54–65. 10.1016/j.ydbio.2014.03.023 24727668

[B8] Bowman A. N. van Amerongen R. Palmer T. D. Nusse R. (2013). Lineage tracing with Axin2 reveals distinct developmental and adult populations of Wnt/beta-catenin-responsive neural stem cells. Proc. Natl. Acad. Sci. U S A 110, 7324–7329. 10.1073/pnas.1305411110 23589866 PMC3645553

[B9] Bramhall N. F. Shi F. Arnold K. Hochedlinger K. Edge A. S. (2014). Lgr5-positive supporting cells generate new hair cells in the postnatal cochlea. Stem Cell Reports 2, 311–322. 10.1016/j.stemcr.2014.01.008 24672754 PMC3964281

[B10] Brignull H. R. Raible D. W. Stone J. S. (2009). Feathers and fins: non-mammalian models for hair cell regeneration. Brain Res. 1277, 12–23. 10.1016/j.brainres.2009.02.028 19245801 PMC2700174

[B11] Brooker R. Hozumi K. Lewis J. (2006). Notch ligands with contrasting functions: Jagged1 and Delta1 in the mouse inner ear. Development 133, 1277–1286. 10.1242/dev.02284 16495313

[B12] Brown S. T. Martin K. Groves A. K. (2003). Molecular basis of inner ear induction. Curr. Top. Dev. Biol. 57, 115–149. 10.1016/s0070-2153(03)57004-1 14674479

[B13] Burns J. C. Cox B. C. Thiede B. R. Zuo J. Corwin J. T. (2012). In vivo proliferative regeneration of balance hair cells in newborn mice. J. Neurosci. 32, 6570–6577. 10.1523/JNEUROSCI.6274-11.2012 22573679 PMC3359838

[B14] Cabrera C. V. Alonso M. C. Johnston P. Phillips R. G. Lawrence P. A. (1987). Phenocopies induced with antisense RNA identify the wingless gene. Cell 50, 659–663. 10.1016/0092-8674(87)90039-0 2440586

[B15] Chai R. Kuo B. Wang T. Liaw E. J. Xia A. Jan T. A.. (2012). Wnt signaling induces proliferation of sensory precursors in the postnatal mouse cochlea. Proc. Natl. Acad. Sci. U S A 109, 8167–8172. 10.1073/pnas.1202774109 22562792 PMC3361451

[B16] Chai R. Xia A. Wang T. Jan T. A. Hayashi T. Bermingham-Mcdonogh O.. (2011). Dynamic expression of Lgr5, a Wnt target gene, in the developing and mature mouse cochlea. J. Assoc. Res. Otolaryngol. 12, 455–469. 10.1007/s10162-011-0267-2 21472479 PMC3123443

[B17] Chang W. Brigande J. V. Fekete D. M. Wu D. K. (2004). The development of semicircular canals in the inner ear: role of FGFs in sensory cristae. Development 131, 4201–4211. 10.1242/dev.01292 15280215

[B18] Clevers H. Loh K. M. Nusse R. (2014). Stem cell signaling. An integral program for tissue renewal and regeneration: Wnt signaling and stem cell control. Science 346:1248012. 10.1126/science.1248012 25278615

[B19] Corwin J. T. Cotanche D. A. (1988). Regeneration of sensory hair cells after acoustic trauma. Science 240, 1772–1774. 10.1126/science.3381100 3381100

[B20] Cox B. C. Chai R. Lenoir A. Liu Z. Zhang L. Nguyen D. H.. (2014). Spontaneous hair cell regeneration in the neonatal mouse cochlea in vivo. Development 141, 816–829. 10.1242/dev.103036 24496619 PMC3912828

[B21] Dabdoub A. Donohue M. J. Brennan A. Wolf V. Montcouquiol M. Sassoon D. A.. (2003). Wnt signaling mediates reorientation of outer hair cell stereociliary bundles in the mammalian cochlea. Development 130, 2375–2384. 10.1242/dev.00448 12702652

[B22] DasGupta R. Fuchs E. (1999). Multiple roles for activated LEF/TCF transcription complexes during hair follicle development and differentiation. Development 126, 4557–4568. 10498690 10.1242/dev.126.20.4557

[B23] de Lau W. Barker N. Low T. Y. Koo B. K. Li V. S. Teunissen H.. (2011). Lgr5 homologues associate with Wnt receptors and mediate R-spondin signalling. Nature 476, 293–297. 10.1038/nature10337 21727895

[B24] de Lau W. B. Snel B. Clevers H. C. (2012). The R-spondin protein family. Genome Biol. 13:242. 10.1186/gb-2012-13-3-242 22439850 PMC3439965

[B25] de Visser K. E. Ciampricotti M. Michalak E. M. Tan D. W. Speksnijder E. N. Hau C. S.. (2012). Developmental stage-specific contribution of LGR5(+) cells to basal and luminal epithelial lineages in the postnatal mammary gland. J. Pathol. 228, 300–309. 10.1002/path.4096 22926799

[B26] Dorsky R. I. Sheldahl L. C. Moon R. T. (2002). A transgenic Lef1/beta-catenin-dependent reporter is expressed in spatially restricted domains throughout zebrafish development. Dev. Biol. 241, 229–237. 10.1006/dbio.2001.0515 11784107

[B27] Duncan L. J. Mangiardi D. A. Matsui J. I. Anderson J. K. Mclaughlin-Williamson K. Cotanche D. A. (2006). Differential expression of unconventional myosins in apoptotic and regenerating chick hair cells confirms two regeneration mechanisms. J. Comp. Neurol. 499, 691–701. 10.1002/cne.21114 17048225 PMC2426907

[B28] Etheridge S. L. Ray S. Li S. Hamblet N. S. Lijam N. Tsang M.. (2008). Murine dishevelled 3 functions in redundant pathways with dishevelled 1 and 2 in normal cardiac outflow tract, cochlea and neural tube development. PLoS Genet. 4:e1000259. 10.1371/journal.pgen.1000259 19008950 PMC2576453

[B29] Ezan J. Montcouquiol M. (2013). Revisiting planar cell polarity in the inner ear. Semin. Cell Dev. Biol. 24, 499–506. 10.1016/j.semcdb.2013.03.012 23562830

[B30] Ferrer-Vaquer A. Piliszek A. Tian G. Aho R. J. Dufort D. Hadjantonakis A. K. (2010). A sensitive and bright single-cell resolution live imaging reporter of Wnt/ss-catenin signaling in the mouse. BMC Dev. Biol. 10:121. 10.1186/1471-213x-10-121 21176145 PMC3017038

[B31] Fevr T. Robine S. Louvard D. Huelsken J. (2007). Wnt/beta-catenin is essential for intestinal homeostasis and maintenance of intestinal stem cells. Mol. Cell. Biol. 27, 7551–7559. 10.1128/mcb.01034-07 17785439 PMC2169070

[B32] Forge A. Li L. Corwin J. T. Nevill G. (1993). Ultrastructural evidence for hair cell regeneration in the mammalian inner ear. Science 259, 1616–1619. 10.1126/science.8456284 8456284

[B33] Forristall C. A. Stellabotte F. Castillo A. Collazo A. (2014). Embryological manipulations in the developing Xenopus inner ear reveal an intrinsic role for Wnt signaling in dorsal-ventral patterning. Dev. Dyn. 243, 1262–1274. 10.1002/dvdy.24116 24500889

[B34] Freter S. Muta Y. Mak S. S. Rinkwitz S. Ladher R. K. (2008). Progressive restriction of otic fate: the role of FGF and Wnt in resolving inner ear potential. Development 135, 3415–3424. 10.1242/dev.026674 18799542

[B35] Freyer L. Morrow B. E. (2010). Canonical Wnt signaling modulates Tbx1, Eya1 and Six1 expression, restricting neurogenesis in the otic vesicle. Dev. Dyn. 239, 1708–1722. 10.1002/dvdy.22308 20503367 PMC2987613

[B36] Gamba L. Cubedo N. Lutfalla G. Ghysen A. Dambly-Chaudiere C. (2010). Lef1 controls patterning and proliferation in the posterior lateral line system of zebrafish. Dev. Dyn. 239, 3163–3171. 10.1002/dvdy.22469 20981829

[B37] Gleason J. E. Szyleyko E. A. Eisenmann D. M. (2006). Multiple redundant Wnt signaling components function in two processes during C. elegans vulval development. Dev. Biol. 298, 442–457. 10.1016/j.ydbio.2006.06.050 16930586

[B38] Golub J. S. Tong L. Ngyuen T. B. Hume C. R. Palmiter R. D. Rubel E. W.. (2012). Hair cell replacement in adult mouse utricles after targeted ablation of hair cells with diphtheria toxin. J. Neurosci. 32, 15093–15105. 10.1523/JNEUROSCI.1709-12.2012 23100430 PMC3544304

[B39] Gómez-Orte E. Sáenz-Narciso B. Moreno S. Cabello J. (2013). Multiple functions of the noncanonical Wnt pathway. Trends Genet. 29, 545–553. 10.1016/j.tig.2013.06.003 23846023

[B40] Harris J. A. Cheng A. G. Cunningham L. L. MacDonald G. Raible D. W. Rubel E. W. (2003). Neomycin-induced hair cell death and rapid regeneration in the lateral line of zebrafish (Danio rerio). J. Assoc. Res. Otolaryngol. 4, 219–234. 10.1007/s10162-002-3022-x 12943374 PMC3202713

[B41] Hayward P. Kalmar T. Arias A. M. (2008). Wnt/Notch signalling and information processing during development. Development 135, 411–424. 10.1242/dev.000505 18192283

[B42] Head J. R. Gacioch L. Pennisi M. Meyers J. R. (2013). Activation of canonical Wnt/beta-catenin signaling stimulates proliferation in neuromasts in the zebrafish posterior lateral line. Dev. Dyn. 242, 832–846. 10.1002/dvdy.23973 23606225

[B43] Hernandez P. P. Olivari F. A. Sarrazin A. F. Sandoval P. C. Allende M. L. (2007). Regeneration in zebrafish lateral line neuromasts: expression of the neural progenitor cell marker sox2 and proliferation-dependent and-independent mechanisms of hair cell renewal. Dev. Neurobiol. 67, 637–654. 10.1002/dneu.20386 17443814

[B44] Huch M. Bonfanti P. Boj S. F. Sato T. Loomans C. J. van de Wetering M.. (2013a). Unlimited in vitro expansion of adult bi-potent pancreas progenitors through the Lgr5/R-spondin axis. EMBO J. 32, 2708–2721. 10.1038/emboj.2013.204 24045232 PMC3801438

[B117] Huch M. Dorrell C. Boj S. F. van Es J. H. Li V. S. van de Wetering M.. (2013b). In vitro expansion of single Lgr5+ liver stem cells induced by Wnt-driven regeneration. Nature 494, 247–250. 10.1038/nature11826 23354049 PMC3634804

[B45] Jacques B. E. Montgomery W. H. Uribe P. M. Yatteau A. Asuncion J. D. Resendiz G.. (2014). The role of Wnt/β-catenin signaling in proliferation and regeneration of the developing basilar papilla and lateral line. Dev. Neurobiol. 74, 438–456. 10.1002/dneu.22134 24115534

[B46] Jacques B. E. Puligilla C. Weichert R. M. Ferrer-Vaquer A. Hadjantonakis A. K. Kelley M. W.. (2012). A dual function for canonical Wnt/β-catenin signaling in the developing mammalian cochlea. Development 139, 4395–4404. 10.1242/dev.080358 23132246 PMC3509733

[B47] Jaks V. Barker N. Kasper M. van Es J. H. Snippert H. J. Clevers H.. (2008). Lgr5 marks cycling, yet long-lived, hair follicle stem cells. Nat. Genet. 40, 1291–1299. 10.1038/ng.239 18849992

[B48] Jan T. A. Chai R. Sayyid Z. N. van Amerongen R. Xia A. Wang T.. (2013). Tympanic border cells are Wnt-responsive and can act as progenitors for postnatal mouse cochlear cells. Development 140, 1196–1206. 10.1242/dev.087528 23444352 PMC3585657

[B49] Jayasena C. S. Ohyama T. Segil N. Groves A. K. (2008). Notch signaling augments the canonical Wnt pathway to specify the size of the otic placode. Development 135, 2251–2261. 10.1242/dev.017905 18495817 PMC2575054

[B50] Jho E. H. Zhang T. Domon C. Joo C. K. Freund J. N. Costantini F. (2002). Wnt/beta-catenin/Tcf signaling induces the transcription of Axin2, a negative regulator of the signaling pathway. Mol. Cell. Biol. 22, 1172–1183. 10.1128/mcb.22.4.1172-1183.2002 11809808 PMC134648

[B51] Jiang L. Romero-Carvajal A. Haug J. S. Seidel C. W. Piotrowski T. (2014). Gene-expression analysis of hair cell regeneration in the zebrafish lateral line. Proc. Natl. Acad. Sci. U S A 111, E1383–E1392. 10.1073/pnas.1402898111 24706903 PMC3986165

[B52] Jin Y. R. Yoon J. K. (2012). The R-spondin family of proteins: emerging regulators of WNT signaling. Int. J. Biochem. Cell Biol. 44, 2278–2287. 10.1016/j.biocel.2012.09.006 22982762 PMC3496018

[B53] Jones J. E. Corwin J. T. (1993). Replacement of lateral line sensory organs during tail regeneration in salamanders: identification of progenitor cells and analysis of leukocyte activity. J. Neurosci. 13, 1022–1034. 8441001 10.1523/JNEUROSCI.13-03-01022.1993 PMC6576617

[B54] Kadowaki T. Wilder E. Klingensmith J. Zachary K. Perrimon N. (1996). The segment polarity gene porcupine encodes a putative multitransmembrane protein involved in wingless processing. Genes Dev. 10, 3116–3128. 10.1101/gad.10.24.3116 8985181

[B55] Karamboulas C. Ailles L. (2013). Developmental signaling pathways in cancer stem cells of solid tumors. Biochim. Biophys. Acta 1830, 2481–2495. 10.1016/j.bbagen.2012.11.008 23196196

[B56] Kiernan A. E. Xu J. Gridley T. (2006). The notch ligand JAG1 is required for sensory progenitor development in the mammalian inner ear. PLoS Genet. 2:e4. 10.1371/journal.pgen.0020004 16410827 PMC1326221

[B57] Korinek V. Barker N. Moerer P. van Donselaar E. Huls G. Peters P. J.. (1998). Depletion of epithelial stem-cell compartments in the small intestine of mice lacking Tcf-4. Nat. Genet. 19, 379–383. 10.1038/1270 9697701

[B58] Ku Y. C. Renaud N. A. Veile R. A. Helms C. Voelker C. C. Warchol M. E.. (2014). The transcriptome of utricle hair cell regeneration in the avian inner ear. J. Neurosci. 34, 3523–3535. 10.1523/JNEUROSCI.2606-13.2014 24599453 PMC3942572

[B59] Ladher R. K. Anakwe K. U. Gurney A. L. Schoenwolf G. C. Francis-West P. H. (2000). Identification of synergistic signals initiating inner ear development. Science 290, 1965–1967. 10.1126/science.290.5498.1965 11110663

[B60] Ladher R. K. O’Neill P. Begbie J. (2010). From shared lineage to distinct functions: the development of the inner ear and epibranchial placodes. Development 137, 1777–1785. 10.1242/dev.040055 20460364

[B61] Leblond C. P. Stevens C. E. (1948). The constant renewal of the intestinal epithelium in the albino rat. Anat. Rec. 100, 357–377. 10.1002/ar.1091000306 18906253

[B62] Lewis J. Davies A. (2002). Planar cell polarity in the inner ear: how do hair cells acquire their oriented structure? J. Neurobiol. 53, 190–201. 10.1002/neu.10124 12382275

[B63] Lim X. Tan S. H. Koh W. L. Chau R. M. Yan K. S. Kuo C. J.. (2013). Interfollicular epidermal stem cells self-renew via autocrine Wnt signaling. Science 342, 1226–1230. 10.1126/science.1239730 24311688 PMC4081860

[B64] Lin V. Golub J. S. Nguyen T. B. Hume C. R. Oesterle E. C. Stone J. S. (2011). Inhibition of notch activity promotes nonmitotic regeneration of hair cells in the adult mouse utricles. J. Neurosci. 31, 15329–15339. 10.1523/JNEUROSCI.2057-11.2011 22031879 PMC3235543

[B65] Logan C. Y. Nusse R. (2004). The Wnt signaling pathway in development and disease. Annu. Rev. Cell Dev. Biol. 20, 781–810. 10.1146/annurev.cellbio.20.010403.113126 15473860

[B66] Lu Z. Corwin J. T. (2008). The influence of glycogen synthase kinase 3 in limiting cell addition in the mammalian ear. Dev. Neurobiol. 68, 1059–1075. 10.1002/dneu.20635 18470861

[B67] Lustig B. Jerchow B. Sachs M. Weiler S. Pietsch T. Karsten U.. (2002). Negative feedback loop of Wnt signaling through upregulation of conductin/axin2 in colorectal and liver tumors. Mol. Cell. Biol. 22, 1184–1193. 10.1128/mcb.22.4.1184-1193.2002 11809809 PMC134640

[B68] Ma E. Y. Rubel E. W. Raible D. W. (2008). Notch signaling regulates the extent of hair cell regeneration in the zebrafish lateral line. J. Neurosci. 28, 2261–2273. 10.1523/JNEUROSCI.4372-07.2008 18305259 PMC6671837

[B69] Mahoney Rogers A. A. Zhang J. Shim K. (2011). Sprouty1 and Sprouty2 limit both the size of the otic placode and hindbrain Wnt8a by antagonizing FGF signaling. Dev. Biol. 353, 94–104. 10.1016/j.ydbio.2011.02.022 21362415 PMC3075364

[B70] Maretto S. Cordenonsi M. Dupont S. Braghetta P. Broccoli V. Hassan A. B.. (2003). Mapping Wnt/β-catenin signaling during mouse development and in colorectal tumors. Proc. Natl. Acad. Sci. U S A 100, 3299–3304. 10.1073/pnas.0434590100 12626757 PMC152286

[B71] Matsui J. I. Oesterle E. C. Stone J. S. Rubel E. W. (2000). Characterization of damage and regeneration in cultured avian utricles. J. Assoc. Res. Otolaryngol. 1, 46–63. 10.1007/s101620010005 11548237 PMC2504559

[B72] McGraw H. F. Drerup C. M. Culbertson M. D. Linbo T. Raible D. W. Nechiporuk A. V. (2011). Lef1 is required for progenitor cell identity in the zebrafish lateral line primordium. Development 138, 3921–3930. 10.1242/dev.062554 21862556 PMC3160089

[B73] Miller J. R. Hocking A. M. Brown J. D. Moon R. T. (1999). Mechanism and function of signal transduction by the Wnt/beta-catenin and Wnt/Ca2+ pathways. Oncogene 18, 7860–7872. 10.1038/sj.onc.1203245 10630639

[B74] Morrison A. Hodgetts C. Gossler A. Hrabé de Angelis M. Lewis J. (1999). Expression of Delta1 and Serrate1 (Jagged1) in the mouse inner ear. Mech. Dev. 84, 169–172. 10.1016/s0925-4773(99)00066-0 10473135

[B75] Mulvaney J. F. Yatteau A. Sun W. W. Jacques B. Takubo K. Suda T.. (2013). Secreted factor R-Spondin 2 is involved in refinement of patterning of the mammalian cochlea. Dev. Dyn. 242, 179–188. 10.1002/dvdy.23908 23192966 PMC3553274

[B76] Nelson W. J. Nusse R. (2004). Convergence of Wnt, beta-catenin and cadherin pathways. Science 303, 1483–1487. 10.1126/science.1094291 15001769 PMC3372896

[B77] Noda T. Oki S. Kitajima K. Harada T. Komune S. Meno C. (2012). Restriction of Wnt signaling in the dorsal otocyst determines semicircular canal formation in the mouse embryo. Dev. Biol. 362, 83–93. 10.1016/j.ydbio.2011.11.019 22166339

[B78] Noramly S. Grainger R. M. (2002). Determination of the embryonic inner ear. J. Neurobiol. 53, 100–128. 10.1002/neu.10131 12382270

[B79] Nusse R. van Ooyen A. Cox D. Fung Y. K. Varmus H. (1984). Mode of proviral activation of a putative mammary oncogene (int-1) on mouse chromosome 15. Nature 307, 131–136. 10.1038/307131a0 6318122

[B80] Oesterle E. C. Campbell S. (2009). Supporting cell characteristics in long-deafened aged mouse ears. J. Assoc. Res. Otolaryngol. 10, 525–544. 10.1007/s10162-009-0183-x 19644644 PMC2774416

[B81] Ohyama T. Mohamed O. A. Taketo M. M. Dufort D. Groves A. K. (2006). Wnt signals mediate a fate decision between otic placode and epidermis. Development 133, 865–875. 10.1242/dev.02271 16452098

[B82] Padanad M. S. Riley B. B. (2011). Pax2/8 proteins coordinate sequential induction of otic and epibranchial placodes through differential regulation of foxi1, sox3 and fgf24. Dev. Biol. 351, 90–98. 10.1016/j.ydbio.2010.12.036 21215261 PMC3039053

[B83] Peng A. W. Salles F. T. Pan B. Ricci A. J. (2011). Integrating the biophysical and molecular mechanisms of auditory hair cell mechanotransduction. Nat. Commun. 2:523. 10.1038/ncomms1533 22045002 PMC3418221

[B84] Phillips B. T. Storch E. M. Lekven A. C. Riley B. B. (2004). A direct role for Fgf but not Wnt in otic placode induction. Development 131, 923–931. 10.1242/dev.00978 14757644

[B85] Qian D. Jones C. Rzadzinska A. Mark S. Zhang X. Steel K. P.. (2007). Wnt5a functions in planar cell polarity regulation in mice. Dev. Biol. 306, 121–133. 10.1016/j.ydbio.2007.03.011 17433286 PMC1978180

[B86] Rakowiecki S. Epstein D. J. (2013). Divergent roles for Wnt/beta-catenin signaling in epithelial maintenance and breakdown during semicircular canal formation. Development 140, 1730–1739. 10.1242/dev.092882 23487315 PMC3621490

[B87] Riccomagno M. M. Takada S. Epstein D. J. (2005). Wnt-dependent regulation of inner ear morphogenesis is balanced by the opposing and supporting roles of Shh. Genes Dev. 19, 1612–1623. 10.1101/gad.1303905 15961523 PMC1172066

[B88] Rida P. C. Chen P. (2009). Line up and listen: planar cell polarity regulation in the mammalian inner ear. Semin. Cell Dev. Biol. 20, 978–985. 10.1016/j.semcdb.2009.02.007 19508855 PMC2796270

[B89] Rijsewijk F. Schuermann M. Wagenaar E. Parren P. Weigel D. Nusse R. (1987). The Drosophila homolog of the mouse mammary oncogene int-1 is identical to the segment polarity gene wingless. Cell 50, 649–657. 10.1016/0092-8674(87)90038-9 3111720

[B90] Ring A. Kim Y. M. Kahn M. (2014). Wnt/catenin signaling in adult stem cell physiology and disease. Stem Cell Rev. 10, 512–525. 10.1007/s12015-014-9515-2 24825509 PMC4294579

[B91] Roberson D. W. Alosi J. A. Cotanche D. A. (2004). Direct transdifferentiation gives rise to the earliest new hair cells in regenerating avian auditory epithelium. J. Neurosci. Res. 78, 461–471. 10.1002/jnr.20271 15372572

[B92] Ryals B. M. Rubel E. W. (1988). Hair cell regeneration after acoustic trauma in adult Coturnix quail. Science 240, 1774–1776. 10.1126/science.3381101 3381101

[B93] Schuijers J. Clevers H. (2012). Adult mammalian stem cells: the role of Wnt, Lgr5 and R-spondins. EMBO J. 31, 2685–2696. 10.1038/emboj.2012.149 22617424 PMC3380219

[B94] Shang J. Cafaro J. Nehmer R. Stone J. (2010). Supporting cell division is not required for regeneration of auditory hair cells after ototoxic injury in vitro. J. Assoc. Res. Otolaryngol. 11, 203–222. 10.1007/s10162-009-0206-7 20165896 PMC2862922

[B95] Shi F. Hu L. Edge A. S. (2013). Generation of hair cells in neonatal mice by beta-catenin overexpression in Lgr5-positive cochlear progenitors. Proc. Natl. Acad. Sci. U S A 110, 13851–13856. 10.1073/pnas.1219952110 23918377 PMC3752268

[B96] Shi F. Hu L. Jacques B. E. Mulvaney J. F. Dabdoub A. Edge A. S. (2014). β-Catenin is required for hair-cell differentiation in the cochlea. J. Neurosci. 34, 6470–6479. 10.1523/JNEUROSCI.4305-13.2014 24806673 PMC4012306

[B97] Shi F. Kempfle J. S. Edge A. S. (2012). Wnt-responsive Lgr5-expressing stem cells are hair cell progenitors in the cochlea. J. Neurosci. 32, 9639–9648. 10.1523/JNEUROSCI.1064-12.2012 22787049 PMC3417821

[B98] Sienknecht U. J. Fekete D. M. (2008). Comprehensive Wnt-related gene expression during cochlear duct development in chicken. J. Comp. Neurol. 510, 378–395. 10.1002/cne.21791 18671253 PMC2566905

[B99] Sienknecht U. J. Fekete D. M. (2009). Mapping of Wnt, frizzled and Wnt inhibitor gene expression domains in the avian otic primordium. J. Comp. Neurol. 517, 751–764. 10.1002/cne.22169 19842206 PMC3004361

[B100] Steiner A. B. Kim T. Cabot V. Hudspeth A. J. (2014). Dynamic gene expression by putative hair-cell progenitors during regeneration in the zebrafish lateral line. Proc. Natl. Acad. Sci. U S A 111, E1393–E1401. 10.1073/pnas.1318692111 24706895 PMC3986164

[B101] Stevens C. B. Davies A. L. Battista S. Lewis J. H. Fekete D. M. (2003). Forced activation of Wnt signaling alters morphogenesis and sensory organ identity in the chicken inner ear. Dev. Biol. 261, 149–164. 10.1016/s0012-1606(03)00297-5 12941626

[B102] Sui L. Bouwens L. Mfopou J. K. (2013). Signaling pathways during maintenance and definitive endoderm differentiation of embryonic stem cells. Int. J. Dev. Biol. 57, 1–12. 10.1387/ijdb.120115ls 23585347

[B103] Urness L. D. Paxton C. N. Wang X. Schoenwolf G. C. Mansour S. L. (2010). FGF signaling regulates otic placode induction and refinement by controlling both ectodermal target genes and hindbrain Wnt8a. Dev. Biol. 340, 595–604. 10.1016/j.ydbio.2010.02.016 20171206 PMC2854211

[B104] Valdivia L. E. Young R. M. Hawkins T. A. Stickney H. L. Cavodeassi F. Schwarz Q.. (2011). Lef1-dependent Wnt/beta-catenin signalling drives the proliferative engine that maintains tissue homeostasis during lateral line development. Development 138, 3931–3941. 10.1242/dev.062695 21862557 PMC3160090

[B105] van Amerongen R. Bowman A. N. Nusse R. (2012). Developmental stage and time dictate the fate of Wnt/beta-catenin-responsive stem cells in the mammary gland. Cell Stem Cell 11, 387–400. 10.1016/j.stem.2012.05.023 22863533 PMC13155203

[B106] van den Heuvel M. Harryman-Samos C. Klingensmith J. Perrimon N. Nusse R. (1993). Mutations in the segment polarity genes wingless and porcupine impair secretion of the wingless protein. EMBO J. 12, 5293–5302. 8262072 10.1002/j.1460-2075.1993.tb06225.x PMC413795

[B107] Vendrell V. Vázquez-Echeverría C. López-Hernández I. Alonso B. D. Martinez S. Pujades C.. (2013). Roles of Wnt8a during formation and patterning of the mouse inner ear. Mech. Dev. 130, 160–168. 10.1016/j.mod.2012.09.009 23041177

[B118] Wang T. Chai R. Kim G. S. Pham N. Jansson L. Nguyen D.-H. (in press). Lgr5+ cells regenerate hair cells via proliferation and direct transdifferentiation in damaged neonatal mouse utricle. 10.1038/ncomms7613 PMC4391285 25849379

[B108] Wang Y. Guo N. Nathans J. (2006). The role of Frizzled3 and Frizzled6 in neural tube closure and in the planar polarity of inner-ear sensory hair cells. J. Neurosci. 26, 2147–2156. 10.1523/jneurosci.4698-05.2005 16495441 PMC6674805

[B109] Wang J. Mark S. Zhang X. Qian D. Yoo S. J. Radde-Gallwitz K.. (2005). Regulation of polarized extension and planar cell polarity in the cochlea by the vertebrate PCP pathway. Nat. Genet. 37, 980–985. 10.1038/ng1622 16116426 PMC1413588

[B110] Warchol M. E. Corwin J. T. (1996). Regenerative proliferation in organ cultures of the avian cochlea: identification of the initial progenitors and determination of the latency of the proliferative response. J. Neurosci. 16, 5466–5477. 8757259 10.1523/JNEUROSCI.16-17-05466.1996 PMC6578879

[B111] Warchol M. E. Lambert P. R. Goldstein B. J. Forge A. Corwin J. T. (1993). Regenerative proliferation in inner ear sensory epithelia from adult guinea pigs and humans. Science 259, 1619–1622. 10.1126/science.8456285 8456285

[B112] Willert K. Brown J. D. Danenberg E. Duncan A. W. Weissman I. L. Reya T.. (2003). Wnt proteins are lipid-modified and can act as stem cell growth factors. Nature 423, 448–452. 10.1038/nature01611 12717451

[B113] Williams J. A. Holder N. (2000). Cell turnover in neuromasts of zebrafish larvae. Hear. Res. 143, 171–181. 10.1016/s0378-5955(00)00039-3 10771194

[B114] Wu D. K. Kelley M. W. (2012). Molecular mechanisms of inner ear development. Cold Spring Harb. Perspect. Biol. 4:a008409. 10.1101/cshperspect.a008409 22855724 PMC3405860

[B115] Yoshida N. Liberman M. C. (1999). Stereociliary anomaly in the guinea pig: effects of hair bundle rotation on cochlear sensitivity. Hear. Res. 131, 29–38. 10.1016/s0378-5955(99)00008-8 10355602

[B116] Zeng Y. A. Nusse R. (2010). Wnt proteins are self-renewal factors for mammary stem cells and promote their long-term expansion in culture. Cell Stem Cell 6, 568–577. 10.1016/j.stem.2010.03.020 20569694 PMC2917779

